# Targeted Antimicrobial Peptides

**DOI:** 10.3389/fimmu.2012.00309

**Published:** 2012-10-05

**Authors:** Marc Devocelle

**Affiliations:** ^1^Department of Pharmaceutical and Medicinal Chemistry, Centre for Synthesis and Chemical Biology, Royal College of Surgeons in IrelandDublin, Ireland

The existence of natural antimicrobial substances, contributing to the mechanisms of host defenses, has been recognized since the late nineteenth century. In 1963, the *in vitro* antibacterial activity of leukocyte extracts was attributed to basic proteins. Since the late 1980s, cationic peptides with antimicrobial properties have been subsequently identified in other host cells and tissues and in virtually every living species (Lehrer, 2004). The properties of these “Nature's antibiotics” and their multiple functions in host defenses of multicellular organisms support the rationale of developing entirely novel peptide-based therapeutics harnessing the effector mechanisms of innate immunity (Hancock and Sahl, 2006). The term antimicrobial peptides covers different forms of natural macromolecules; ribosomally synthesized and non-post translationally modified innate immunity peptides, or their synthetic analogs, are predominantly considered here. Their antimicrobial and immunomodulatory activities will not be dissociated in general and they will be indistinctively described as (cationic) antimicrobial or host defense peptides.

The main assets of innate immunity peptides originate from their primary activity essentially directed at a universal non-protein target, the bacterial membrane, reinforced by a polypharmacology which includes multiple immunomodulatory and anti-inflammatory activities (Zasloff, 2002; Finlay and Hancock, 2004). Other distinctive and attractive properties in a clinical context comprise their low susceptibility to classical mechanisms of drug resistance, associated with a low propensity to select resistant mutants, their ability to reduce both biofilm and planktonic bacterial counts and to interact with dividing and non-dividing cells by generally targeting a conserved structure which is independent of the proliferative status of the cells (Peschel and Sahl, 2006; Chen et al., 2011; Fjell et al., 2012). Although some mechanisms of bacterial resistance to antimicrobial peptides have been identified, their emergence occurs at significantly lower frequencies than for traditional antibiotics (Peschel and Sahl, 2006). Furthermore, host defense peptides can also act synergistically with these classical antibiotic agents (Giacometti et al., 2000).

Innate immunity peptides are therefore a prospective source of antibiotic candidates with extended clinical lifetimes. They have not been approved for clinical use to date, but have progressed toward commercial development through recent technological advances. About 1,200 to 1,700 antimicrobial peptide sequences have been identified and/or predicted to date. Approximately 15 different peptide-based therapeutic agents are currently in clinical trials for anti-infective and/or anti-inflammatory indications, generally limited to topical administration (Fjell et al., 2012; Yount and Yeaman, 2012). The challenges traditionally associated with the clinical development of host defense peptide candidates for systemic therapies require notably solutions addressing the question of possible toxicity. Owing to their rapid metabolic degradation and/or excretion, high doses of these peptides might be required to maintain therapeutic levels *in vivo*. This may correlate with an improper margin of safety, despite their selectivity for bacterial over mammalian cells (Zasloff, 2002). In addition, the issue of potential immunogenicity should be considered (Mader and Hoskin, 2006). Other concerns may be raised, either inherent to all peptide-based drug candidates, such as low oral bioavailabilities and elevated cost of production, or specific to host defense peptides, in particular their complex pharmacology which could result in uncontrolled off-target toxicity (Pauletti et al., 1997; Hancock and Sahl., 2006; Brown et al., 2007).

Peptide therapeutics are capturing an increasing fraction of the global pharmaceutical pipeline. Advances in peptide modification, formulation, and delivery technologies can overcome some of their pharmacokinetic, bioavailability, and toxicity shortcomings and likewise have been applied to innate immunity peptides. The latter have been modified by either optimizing the length and content of their sequences, to increase their selective antibacterial activity, or by conversion into peptidomimetics, to improve their pharmacokinetic properties. In the first case, minimizing the length of the sequence and systematically substituting each residue with other coded amino acids, can yield peptide candidates with improved antibacterial activity and/or increased activity differentials between prokaryotic and eukaryotic (generally erythrocytes) cells. This work has been performed in parallel with structure activity relationship studies for the rationale design of therapeutic candidates (Fjell et al., 2012). It focused on the direct antibacterial activity of some selected candidates, but immunomodulatory peptides devoid of *in vitro* antimicrobial activity have also been optimized (Hancock et al., 2012). In the second case, peptidomimetics, structures departing from the traditional peptide backbone and/or stereochemistry but reproducing the biological activity of the parent sequence, have been generated as candidates resistant to proteolysis. They include sequences assembled from non-natural amino acids (e.g., d- or β-amino acids or proteinogenic amino acid analogs with increased hydrophobicity), N- or C-terminally modified with lipophilic chains or groups and also peptoids and non-peptide mimetics (e.g., aminosteroids or amphiphilic polymers; Brogden and Brogden, 2011; Yount and Yeaman, 2012). Other methods for improving the pharmacokinetic properties, preventing the immunogenicity and serum protein inactivation of biopharmaceuticals, such as the pegylation technology, have also been implemented (Harris and Chess, 2003; Imura et al., 2007).

The issue of unknown toxicology for systemically administered innate immunity peptides has also been addressed in a number of these approaches. Alternatively, some modified peptides or peptidomimetics may retain not only the activity, but also the toxicity of the parent peptide. To efficiently and reliably control the toxicity of a therapeutic candidate, a selective delivery technology can be implemented as an alternative method, or in combination with the previous approaches. These methods can confine the activity of innate immunity peptides to the sites of infection and generate thereby targeted antimicrobial peptides. They consist either of peptide sequences conjugated to targeting moieties, modified as inactive precursors which can be selectively activated at a target body site, or loaded in drug delivery systems that can be targeted to their desired site of action. In the former case, the targeting moiety can be an antibody directed against a pathogen-specific antigen. A host defense peptide sequence is in this case conjugated to the sequence of an antibody fragment through the production of a fusion protein containing or not a cleavable linker between the targeting and antimicrobial domains (Peschen et al., 2004; Szynol et al., 2006; Franzman et al., 2009). The resulting immunoconjugate can, for example, confer specific resistance to a fungus in transgenic plants or discriminate a specific periodontal pathogen from other bacteria of the oral commensal flora. The targeting moiety can also be another peptide sequence that can bind selectively to a specific cell surface receptor of a bacterial pathogen, such as a pheromone receptor for instance. A targeted antimicrobial candidate is then generated as a fusion or synthetic peptide comprising the antimicrobial and pheromone sequences (Qiu et al., 2003; Eckert et al., 2006). These chimeric peptides can, for example, discriminate between *Staphylococcus aureus* (MSSA and MRSA strains) and *Staphylococcus epidermidis* or *Streptococcus pneumoniae* and be protective in a mouse model of infection with MRSA, or selectively eliminate the cariogenic bacterium *Streptococcus mutans* in planktonic cultures and multispecies biofilms, without affecting closely related non-cariogenic oral streptococci.

The generation of targeted antimicrobial peptides has also been investigated through the conjugation of a classical antibiotic to a host defense peptide sequence, to increase its selectivity and activity against bacteria expressing the target of the conventional antibiotic. Vancomycin-peptide conjugates of magainin 2 were for example constructed by using the copper(I)-catalyzed azide-alkyne cyclo-addition (Arnusch et al., 2012). This approach yields hybrid antibiotics containing 2 different pharmacophores, which can capitalize on their dual activity to increase their efficiency and delay resistance development, but can also restore the activity of the classical agent against resistant bacteria (Pokrovskaya and Baasov, 2010). Another application of these hybrid antibiotics is in the generation of antimicrobial peptide prodrug candidates, where the classical antibiotic acts as a promoiety rather than as an active agent (Rautio et al., 2008). For example, conjugation of a cephalosporin to a host defense peptide sequence can reversibly modulate one of the activity determinants, the net charge, of the parent peptide (Desgranges et al., 2012). The latter can be selectively released from the conjugate by β-lactamase-mediated hydrolysis of the cephalosporin's lactam ring, a reaction which constitutes the main mechanism of antibiotic resistance in Gram-negative pathogens. A prodrug modification has been proposed as a promising strategy to potentiate the systemic applications of host defense peptides and has been frequently used to overcome the toxicity of low molecular weight drug candidates, but also of the lipopeptide polymyxin E (Hancock, 2001; Stella, 2004). Nature also selected a prodrug approach to regulate and control the activity of some innate immunity peptides (Yeaman and Yount, 2007). This natural mechanism can be synthetically mimicked by generating peptide sequences containing three domains, including an oligo-glutamyl profragment, a linker cleavable by a target disease-associated protease and the parent sequence of a host defense peptide assembled from d-amino acids (Desgranges et al., 2011). Activity and toxicity differentials can be achieved between a neutrophil elastase-dependent propeptide and its parent peptide, although enzymes of bacterial origin that have narrow substrate specificities and no mammalian homologs might have to be targeted for the activation of a propeptide systemically administered. Finally, peptide prodrugs containing a promoiety yielding itself a pharmacologically active entity upon activation, i.e., co-drugs, can target an antimicrobial peptide to a site of bacterial infection or colonization while allowing the co-delivery of an agent with a complementary activity. For example, conjugation through an azo bond of aniline-based agents such as 4-aminophenylacetic acid or 5-aminosalicylic acid, to the N-terminus of a peptide requiring a free *N*^〈^-amino group, can generate co-drug candidates for colonic delivery of non-steroidal anti-inflammatory and antibiotic agents (Kennedy et al., 2011). The metabolic activation of these two therapeutic candidates can only be performed by azo-reductases, enzymes limited to anaerobic bacteria only, restricting thereby the activity of the peptide to the colon. Alternatively, environmentally sensitive antimicrobial peptides, such as pH-responsive sequences, can inherently have their activity confined to a particular body site (Li et al., 2010).

Antimicrobial peptides can also be targeted through their loading in nanoparticulate systems with selective delivery capacity. They include liposomes, including stealth liposomes, polymeric structures, including hydrogels and dendritic polymers, nanospheres, and nanocapsules, carbon nanotubes and DNA cages (Urbán et al., 2012). Their nanoscale size determines their drug loading capacities, but prolongs their circulation times. Their structure can protect the cargo from metabolic degradation and limit its toxicity by preventing its interaction with plasma proteins and host cell surfaces. Furthermore, the release of the cargo can be environmentally controlled or the surfaces of these nano-carriers can be modified with targeting moieties to allow their selective delivery to specific cells or tissues and even through the blood-brain barrier. Some of these drug delivery systems (e.g., liposome, protein, and polymer carriers) have already been investigated with host defense peptides as cargoes (McClanahan et al., 2011; Yount and Yeaman, 2012).

Finally, the concern related to the elevated cost of production of these candidates is now moderated by the advances in their production methods, such as the recombinant expression in heterologous microbial systems (Mygind et al., 2005; Li, 2011). Applicable to the production of peptides assembled from natural amino acids, it is also complemented by the solid phase synthetic approach which allows the cost-effective production on a multi-tonne per year scale of peptides, but also modified peptides and peptidomimetics, that can meet the requirements of regulatory agencies (Bray, 2003).

The therapeutic potential of host defense peptides can be extended beyond the anti-infective and anti-inflammatory applications to cancer therapy. Indeed, innate immunity peptides can be active against prokaryotic and neoplastic eukaryotic cells, according to the high anionic lipid content of the bacterial, malignant cells and mitochondrial membranes and to structural differences between the bacterial or cancer cell membranes and the plasma membrane of normal eukaryotic cells (Papo and Shai, 2005; Mader and Hoskin, 2006). Some of the approaches developed in the anti-infective and anticancer areas to address, separately or collectively, the limitations of antimicrobial peptides, could address the clinical shortcomings associated with these candidates and the optimization and/or combination of these approaches, together with the advances in production and purification methods, could ultimately realize the full therapeutic potential of antimicrobial peptides.
